# Distance to hospital and socioeconomic status influence secondary health care use

**DOI:** 10.3109/02813432.2012.759712

**Published:** 2013-06

**Authors:** Andrzej Zielinski, Lars Borgquist, Anders Halling

**Affiliations:** ^1^Blekinge Centre of Competence, Karlskrona, Sweden; ^2^Linköping University, Department of Medical and Health Sciences, General Practice, Linköping, Sweden; ^3^Research Unit for General Practice, Institute of Public Health, University of Southern Denmark, Odense, Denmark; ^4^Lund University, Department of Clinical Sciences in Malmö, General Practice/Family Medicine, Malmö, Sweden

**Keywords:** Case-mix, comorbidity, general practice, geographical distance, health care utilization, socioeconomic status, Sweden

## Abstract

**Objective:**

The aim of this study was to investigate how distance to hospital and socioeconomic status (SES) influence the use of secondary health care (SHC) when taking comorbidity into account.

**Design and setting:**

A register-based study in Östergötland County.

**Subjects:**

The adult population of Östergötland County.

**Main outcome measures:**

Odds of SHC use in the population and rates of SHC use by patients were studied after taking into account comorbidity level assigned using the Adjusted Clinical Groups (ACG) Case-Mix System. The baseline for analysis of SES was individuals with the lowest education level (level 1) and the lowest income (1st quartile).

**Results:**

The study showed both positive and negative association between SES and use of SHC. The risk of incurring SHC costs was 12% higher for individuals with education level 1. Individuals with income in the 2nd quartile had a 4% higher risk of incurring SHC costs but a 17% lower risk of emergency department visits. Individuals with income in the 4th quartile had 9% lower risk of hospitalization. The risk of using SHC services for the population was not associated with distance to hospital. Patients living over 40 km from hospital and patients with higher SES had lower use of SHC services.

**Conclusions:**

It was found that distance to hospital and SES influence SHC use after adjusting for comorbidity level, age, and gender. These results suggest that GPs and health care managers should pay a higher degree of attention to this when planning primary care services in order to minimize the potentially redundant use of SHC.

There is evidence that distance to hospital and socioeconomic status (SES) influence secondary health care (SHC) use.

- The association between distance to hospital, SES, and the use of SHC was investigated in a population of about 400 000 individuals.
- Shorter distance and both higher and lower SES were associated with higher use of SHC services after adjustment for comorbidity, age, and gender.
- GPs and health care managers should be aware of these differences in SHC use in the population in order to reach individuals with potentially redundant SHC use.

## Introduction

Primary care is the base of the Swedish health care system and has the important function of providing easy access to health care and satisfying basic health care needs. Although most patients’ problems can be solved in primary care, there are some cases that need referral to secondary health care (SHC) for more sophisticated diagnosis and treatment. However, SHC receives some patients who do not need specialist care, which exposes patients to unnecessary diagnosis and increasing health care costs. It is important to identify and provide better access to primary care for such patients. Comorbidity is an important factor for the use of SHC [1] and is also associated with an increased number of visits to SHC [2] and higher health care expenditure [3]. Ideally, comorbidity alone should be the factor of importance for use of SHC. However, there are also other factors such as gender and age [4,5], geographical distance to specialist clinics [6], or low socioeconomic status (SES) [7] that can influence referral rates to SHC and hospitalizations.

How geographical distance to hospital and SES influence the use of SHC has been studied previously, but in this population-based study the comorbidity level of the patients is taken into account, which has not previously been done in Sweden. The aim of this study was to examine the importance of these factors after adjusting for comorbidity.

## Material and methods

### Study design and population

The population of this study consisted of people over age 19 living in Östergötland County, which had about 400 000 inhabitants in 2006. Data were obtained from the Care Data Warehouse in Östergötland [8]. This data register collects information about health care visits sent monthly from all public and private health care units in the county.

Referrals were generally required for appointment to specialists in three studied hospitals where 90–95% of SHC in Östergötland took place.

Visits to emergency department, total SHC costs, and hospitalization days during 2006 were the outcome variables for studying the use of SHC. The independent variables were comorbidity, gender, age, distance to hospital, educational level, and disposable income.

Our study consisted of two parts with analysis of three dependent variables. In the first part we analysed the risk of SHC use by all individuals in the population. In the other part the use of SHC only by those individuals who had in fact used SHC, i.e. patients, was analysed.

All primary care and SHC diagnoses received in 2006 were used in the analysis of comorbidity level by means of the Adjusted Clinical Groups Case-Mix System (ACG) 7.1 [9]. Each of the individuals in the population was assigned to one of six levels of comorbidity, known as resource utilization bands (RUB). RUB 0 meant the lowest and RUB 5 the highest comorbidity level. The ACG has been previously evaluated in other studies [10–12], including in Sweden [13,14].

Distance to hospital was divided into three intervals: up to 19, between 20 and 39, and over 39 km.

Information on individual level of education and disposable income, i.e. two socioeconomic factors, was obtained from Statistics Sweden. We excluded people under 20 years old due to incomplete records of education and lack of or very low income. Because of the significant lack of information on education, individuals aged 70 and above were excluded when we analysed the effect of educational level.

Education was divided into four levels:

- Level 1: Education below 9 years.
- Level 2: Education 9–10 years.
- Level 3: Education 11–12 years.
- Level 4: Education over 12 years.

Income was measured as a continuous variable and divided into four quartiles with individuals with the lowest income in the 1st quartile and with the highest in the 4th quartile.

### Statistical analysis

STATA version 10 (Stata Corporation, Texas, USA) was used for the statistical analyses. To study the independent variables in relation to each other, multivariate statistics were used. After adjustment for comorbidity, gender, and age, the effect of distance to hospital, educational level, or income level on dependent variables was analysed in regression analysis. Due to the large proportion of the population without SHC use, Poisson regression analysis was not considered valid. The best statistical analysis that could model our data was considered to be zero- inflated negative binominal regression [15], which performs two simultaneous analyses. One analysis, similar to logistic regression, answers the question of what the odds in the studied population are of belonging to the group without SHC use and gives an odds ratio (OR). Inversed ORs show odds of using SHC. The other analysis, similar to Poisson regression, shows the effect of increasing the independent variable by one unit among those who were SHC patients and gives an incidence rate ratio (IRR). Individuals and patients living nearest to hospital, with the lowest education (level 1) and income (1st quartile), were the baseline in regression analysis.

## Results

Among 313 982 individuals included in this study 12% had emergency department visits, 46% incurred SHC costs, and 10% were hospitalized. A higher number of emergency department visits, SHC costs, and number of hospitalization days were all associated with female gender, higher age, higher comorbidity level, shorter distance to hospital, and lower education or income level (Table I).

**Table I.** Use of secondary health care by patients in the studied population (percentage of all individuals in the population with the same variable).

	Emergency visit		Secondary health care costs		Hospitalization	
	n	(%)	n	(%)	n	(%)
All	38 058	(12.1)	145 880	(46.5)	33 111	(10.5)
Gender:						
Women	19 545	(12.3)	88 777	(55.9)	19 463	(12.3)
Men	18 512	(11.9)	57 099	(36.8)	13 644	(8.8)
Age:						
20–39	9399	(9.5)	44 186	(44.4)	7832	(7.9)
40–59	10 165	(9.4)	43 854	(40.3)	6844	(6.3)
60–79	11 554	(14.5)	40 874	(51.2)	11 144	(14.0)
80–	6939	(26.7)	16 962	(65.2)	7287	(28.0)
Comorbidity:						
RUB 0	186	(0.2)	1379	(1.4)	5	(0.0)
RUB 1	2969	(6.8)	24 033	(54.8)	533	(1.2)
RUB 2	7815	(12.1)	35 544	(55.0)	3384	(5.2)
RUB 3	18 625	(20.8)	71 298	(79.6)	19 525	(21.8)
RUB 4	6079	(55.8)	10 433	(95.7)	6853	(62.9)
RUB 5	2383	(74.1)	3189	(99.2)	2807	(87.3)
Distance to hospital:						
0–19 km	29 137	(12.8)	107 499	(47.2)	24 384	(10.7)
20–39 km	5080	(10.9)	21 127	(45.3)	4779	(10.3)
> 40 km	2156	(9.0)	9907	(41.5)	2320	(9.7)
Missing	1685	(10.6)	7347	(46.0)	1628	(10.2)
Education level:^1^						
1	2839	(12.6)	10 339	(46.0)	2432	(10.8)
2	3250	(12.3)	11 850	(44.7)	2347	(8.9)
3	12 716	(10.0)	54 603	(43.1)	9458	(7.5)
4	6406	(7.9)	33 955	(42.0)	5439	(6.7)
Missing	725	(23.5)	1661	(53.8)	822	(26.6)
Income quartile:						
1	11 726	(14.9)	40 148	(51.2)	11 386	(14.5)
2	11 483	(14.6)	42 657	(54.4)	11 204	(14.3)
3	7805	(9.9)	34 903	(44.5)	5878	(7.5)
4	7043	(9.0)	28 166	(35.9)	4 639	(5.9)
Missing	1	(0.5)	6	(3.0)	4	(1.9)

n = number; RUB = resource utilizations band; km = kilometre; ^1^education level for individuals under age 70; education level 1 means the lowest education; income quartile 1 the lowest income.

### Individuals in the population

No statistical differences in odds of SHC use and distance to hospital were found in the population after adjustment for comorbidity level, gender, and age (Table II). In contrast, SES influenced odds of SHC use. Individuals with education level 4 had 12% higher odds of incurring SHC costs. Having an income in the 2nd quartile was associated with 17% lower risk of visit to the emergency department, but on the other hand a 4% higher risk of incurring SHC costs. Income in the 3rd and 4th quartiles meant a 19–21% lower risk of being hospitalized (Table II).

**Table II.** Odds of receiving secondary health care in the studied population, adjusted for comorbidity, gender, and age.

	Emergency visit	Secondary health care costs	Days in hospital
	OR (CI)	OR (CI)	OR (CI)
Distance to the hospital:			
0–19 km	1.00	1.00	1.00
20–39 km	0.96 (0.63–1.47)	0.92 (0.75–1.12)	1.01 (0.86–1.19)
over 40 km	0.71 (0.40–1.25)	0.73 (0.52–1.02)	0.86 (0.66–1.12)
Education level:			
1	1.00	1.00	
2	1.24 (0.85–1.82)	1.05 (0.99–1.12)	
3	0.82 (0.60–1.12)	1.03 (0.98–1.09)	
4	0.77 (0.55–1.08)	1.12** (1.05–1.19)	
Income quartile:			
1	1.00	1.00	1.00
2	0.83** (0.75–0.92)	1.04* (1.01–1.09)	1.04 (0.98–1.11)
3	0.97 (0.85–1.11)	1.00 (0.96–1.04)	0.89* (0.83–0.96)
4	0.99 (0.82–1.21)	0.96 (0.92–1.02)	0.91* (0.83–0.99)

OR = odds ratio; CI = confidence interval; km = kilometre; *p < 0.05, **p < 0.001.

### Patients

SHC patients living over 40 km from hospital incurred 9% lower SHC costs and number of hospitalization days compared with those living closest to hospital (Table III).

**Table III.** Rate ratios of secondary health care use by patients, adjusted for comorbidity, gender, and age.

	Emergency visit	Secondary health care costs	Days in hospital
	IRR (CI)	IRR (CI)	IRR (CI)
Distance to the hospital:			
0–19 km	1.00	1.00	1.00
20–39 km	0.81 (0.64–1.06)	0.99 (0.93–1.04)	0.95 (0.88–1.03)
over 40 km	0.71 (0.48–1.04)	0.91*** (0.88–0.94)	0.91* (0.84–0.98)
Education level:			
1	1.00	1.00	
2	1.03 (0.96–1.11)	1.16*** (1.08–1.24)	
3	0.98 (0.92–1.04)	1.01 (0.95–1.06)	
4	0.87* (0.78–0.98)	0.95 (0.90–1.01)	
Income quartile:			
1	1.00	1.00	1.00
2	0.99 (0.95–1.04)	0.92*** (0.89–0.96)	0.83* (0.78–0.88)
3	0.84***	0.78*** (0.78–0.90)	0.66* (0.75–0.82)
4	0.86**	0.78*** (0.79–0.95)	0.66* (0.75–0.81)

IRR = incidence rate ratio; CI = confidence interval; km = kilometre; *p < 0.05, **p < 0.01, ***p < 0.001.

Patients with education level 2 incurred 16% higher SHC costs, but patients with education level 4 had 13% fewer visits to the emergency department. Emergency department visits, SHC costs, and hospitalization days decreased significantly with increasing patient income (Table III).

## Discussion

### Principal findings

In this study we examined both risk of SHC use in the whole population and SHC use by the part of the population actually using SHC. High association between comorbidity and low SES has been seen before [16–18]. We adjusted for comorbidity in order to see whether closeness to hospital or SES level affected SHC utilization independently of comorbidity. We found that taking comorbidity, age, and gender into account changed, but did not remove, the variability in the use of SHC influenced by distance to hospital and SES.

Patients living farthest from the hospital had lower SHC use, but not fewer visits to the emergency department. Generally, the higher income the patient had, the lower the rate of SHC use. It was interesting, however, that individuals with education level 4 but not with income in the 4th quartile had a higher risk of incurring SHC costs.

### Strengths and weaknesses of the study

A population-based study analysing the influence of distance to hospital and SES on utilization of SHC after adjustment for comorbidity has not previously been carried out in Sweden. Analysis of SES factors and comorbidity at an individual level is the strength of this study. Our data were based on electronic patient records from primary care and SHC, ensuring that all SHC contacts by adults in the population during 2006 were included. The ACG system uses diagnoses to assess comorbidity. Therefore the quality of registration of diagnoses is an important issue [12]. The diagnoses in our study were not validated. However, prior studies in Sweden have presented data where up to 75% of all inhabitants in the studied population and about 90% of encounters had registered diagnoses [19]. Because ACG was not used for reimbursement in the county in 2006, the probability of up-coding is unlikely. ACG has previously been shown as a useful tool to measure and study comorbidity [12,20,21].

Exclusion of individuals aged 70 and above from analysis of the education variable due to lack of data on education was a weakness. It can be a potential cause of bias, as the older part of population can differ from the younger ones in education level, and it can explain relatively small differences in SHC use between groups with various education levels. Another limitation is that income can be very low during the education period for young adults and then it can increase very quickly when they finish their studies. Thus income level is not a good indicator of SES in this group. Another problem was the zero-inflated negative binominal regression analysis using the STATA program, which employs an iterative process in successive steps to calculate the estimates. Although we ran the analysis for several hours it was impossible to carry out when we tried to analyse the education variable and hospitalization days, as the program went into an infinite loop. For this reason it was not possible to analyse how much hospitalization days were influenced by education level.

### Previous work

As in our study, longer distance to SHC was associated in prior studies with lower use of SHC [6] and living in an urban location with higher utilization of SHC [22]. In a study from Germany patients living in rural areas had a significantly lower number of visits to SHC than those living in an urban location [23]. One explanation may be that doctors working in rural areas may have a lower tendency to refer to specialists [24]. However, in those studies comorbidity level was not taken into consideration. An interesting result in our study is that the risk of using SHC in the population was not significantly associated with the distance between hospital and the individual's place of residence, although frequency of SHC use was lower in patients living farthest from the hospital.

In the study from Canada, high-educated individuals with the same comorbidity level were more likely to see specialists and to bypass primary care [22]. However, no income-related differences in SHC use after adjustment for other variables were seen, while our results show that income was an important factor for SHC use. Among patients with the same comorbidity level, higher income (3rd and 4th quartiles) was associated with significantly lower use of SHC. An association between low education and higher use of health care services after adjustment for comorbidity [25] and preference for direct access to specialists among highly educated individuals has been observed before [26]. In our studied population the risk of having SHC costs was higher for individuals with education level 4, which may mean higher demands for specialist care or better access to SHC. On the other hand, we can see that SHC costs were higher for patients with education level 2. The question is whether the discrepancy is due to differences in health awareness, or access to information or SHC between people with different education levels. More frequent emergency department visits by patients with education level 2 suggests that they contact SHC when symptoms are acute, probably in a more advanced phase of the illness, which can increase SHC costs.

The use of SHC services by patients with income in the 4th quartile was significantly lower than for those in the 1st quartile, which is different from results from the Baltic countries, where higher income was associated with higher SHC utilization [27]. This shows that different indicators of SES play different roles in SHC use.

## Conclusion

We found that geographical distance to hospital and SES influence the use of SHC, both in the population and among patients, when controlling for comorbidity level, gender, and age. Taking comorbidity into account gave the possibility to see the effect and importance of these studied factors. We believe that understanding the use of SHC by patients could result in more adequate allocation of resources in primary care and a decrease in potentially redundant SHC use.

## Ethics

The study was approved by the Research Ethics Committee at Linköping University.
